# Development over time in point-of-care test use in Danish daytime and out-of-hours general practice: a register-based study

**DOI:** 10.1080/02813432.2023.2187667

**Published:** 2023-03-20

**Authors:** Niels Kjær, Malene Plejdrup Hansen, Henrik Schou Pedersen, Morten Bondo Christensen, Linda Huibers

**Affiliations:** a Research Unit for General Practice, Aarhus, Denmark; b Center for General Practice at, Aalborg University, Aalborg, Denmark; c Institute for Public Health, Aarhus University, Aarhus, Denmark

**Keywords:** Point-of-care testing, C-reactive protein, general practice, after-hours care, out-of-hours medical care

## Abstract

**Objective:**

To describe the development over time of the use of C-reactive protein (CRP) and rapid streptococcal detection test (RADT) point-of-care tests (POCT) in Danish general practice and to explore associations between patient characteristics and POCT use (i.e. CRP and RADT).

**Design and settings:**

A register-based study including all general practice clinic consultations in daytime and out-of-hours (OOH) settings in Denmark between 2003 and 2018.

**Subjects:**

All citizens who had at least one clinic consultation in daytime or OOH general practice within the study period.

**Main outcome measures:**

We estimated the total and relative use of CRP and RADT POCTs and described the development over time. Crude and adjusted proportion ratios (PRs) were calculated to explore associations between patient characteristics and POCT use.

**Results:**

Overall, the relative use of CRP POCTs increased. At OOH, a steep increase was noticed around 2012. The relative use of RADT decreased. Patient age 40–59 years and existing comorbidity were significantly associated with a higher use of CRP testing in both settings. A significantly lower use of CRP testing was found for patients with higher educational level. We found a significantly higher use of RADT testing for patients aged 0–19 years and with higher household educational level, whereas comorbidity was associated with a lower use of RADT testing.

**Conclusion:**

The use of CRP POCT increased over time, whereas the use of RADT POCT decreased. Perhaps the success of implementing CRP as a tool for reducing antibiotic use has reached it limit. Future studies should focus on how and when POCT are used most optimal.

**Key points**
- CRP POC tests and RADT POCTs are frequently used diagnostic tools in general practice, both in daytime and in the out-of-hours setting.
- There was an increased use of CRP POCTs, particularly in out-of-hours general practice, whereas the use of RADT POCTs declined between 2003 and 2018.
- CRP POCTs were associated with age of 40–59 years and co-morbidity, while the use of RADT was mostly associated with younger age.

## Introduction

Infections are a common cause of serious illness worldwide [1,2]. A substantial part of contacts with general practice concerns symptoms related to infections, in particular outside office hours [3–5]. In case of a bacterial infection, antibiotic treatment can be indicated. Antibiotic prescribing patterns vary significantly [6–9], and excessive use contributes to the increasing problems with antimicrobial resistance [10]. The last decades, several point-of-care tests (POCTs), such as C-reactive protein (CRP) and Rapid streptococcal detection test (RADT), have been introduced to perform on site testing in case of suspected infections. CRP and RADT POCTs aim to support general practitioners (GPs) to identify patients who will benefit from antibiotic treatment [11,12], thereby reducing diagnostic uncertainty and contributing to prudent use of antibiotics [9,11,13–15]. A recent study found that patient age, sociodemographic factors, and comorbidity influence the decision to perform a CRP test in Danish general practice [16].

Patients with acute infections can contact general practice during daytime and out-of-hours (OOH); in both settings GPs could use a POCT. The availability and use of POCTs in general practice varies considerably between countries [4,9,17–20]. A study comparing GPs in Australia, Belgium, the Netherlands, United Kingdom, and the United States found country variations in RADT and CRP POCT use between 1–15% and 3–48% respectively [21].

OOH general practice differs from daytime care, for example by having a higher prevalence of patients with acute infections attending, patients often being unknown to the GP, and limited access to POCTs. According to a Norwegian study, more CRP POCTs were used in consultations with 0- to 5-year-old children in OOH (44%) than in daytime (31%) in 2009–2011 [4]. A Danish study found that the use of CRP POCTs in daytime general practice increased from 2004 to 2013, whereas RADT POCT decreased [20]. However, the use of POCTs at Danish OOH general practice services remains unclear. As daytime and OOH general practice differ and the implementation of POCTs in OOH was more difficult, exploration of the development over time of POCT use in both settings is relevant. Thus, we aimed to describe the development over time of the use of CRP- and RADT POCTs in Danish daytime and OOH general practices from 2003 to 2018. Furthermore, we aimed to explore associations between patient characteristics and POCT use (i.e. CRP and RADT).

## Methods

### Study design and setting

We conducted a register-based study of CRP POCTs and RADT POCTs use in Danish general practice, both in daytime practices and at OOH services. The study population consisted of all citizens who had at least one clinic consultation with a daytime general practice or OOH general practice service in one of the five Danish regions from 2003 to 2018.

Services provided by Danish general practice is tax-funded and free of charge for the patient. Almost all Danish citizens are listed with a general practice. GPs act as gatekeepers to secondary care. Patients can contact their own GP during daytime or the OOH general practice service (i.e. GP cooperative) outside office hours. GP cooperatives exist in four out of five regions and are open between 4 pm to 8am from Monday to Friday, all weekends, and bank holidays. GPs answer patients calls, perform telephone triage, and either provide a telephone consultation or refer patients to a clinic consultation or home visit [22]. Nurses have been increasingly introduced to support GPs in the work at the clinical consultations but are still performing fewer tasks in OOH services compared to daytime practice. In 2014, the Capital Region of Denmark introduced the medical helpline 1813 (MH-1813) to provide OOH care. At MH 1813, nurses and physicians perform telephone triage, and patients in need of a clinic consultation are referred to a hospital setting [22]. Thus, for the Capital Region of Denmark, we only included OOH consultations until 31-12-2013.

Danish GPs are paid by fee-for-service, including a fee for conducting CRP and RADT POCTs (in 2022 approximately 10 euro and 8 euro respectively) [23,24]. Daytime practices have varying organisations, with some practices having nurses and other staff providing own consultations for patients with symptoms and signs of an acute infection and/or performing POCTs prior to a GP consultation [25]. CRP and RADT POCTs are available in all Danish daytime practices and performed either by practice staff or by GPs. When CRP and RADT POCTs were first introduced in Denmark, GPs had to bring POCTs to the OOH services themselves. However, in recent years, first RADT and later CRP POCTs became available in the OOH consultations rooms. GPs use provider identification numbers to get reimbursement. In daytime practice, these provider identification numbers often include several GPs who work in the same practice. Outside office hours, the GPs working in daytime mostly use their daytime provider identification number, whereas doctors who only do OOH work have specific provider identification numbers.

### Outcome measures

To describe the use over time, we reported the total number of CRP and RADT POCTs and the number of POCTs per 1,000 clinic consultations.

### Data collection

Data were obtained from the Danish national registers. The National Health Insurance Service Register (NHISR) provided the unique patient identification number, provider identification number, date and time of contact, type of contact, region, CRP POCT use, and RADT POCT use [26]. The Danish Civil Registration System provided data on patient characteristics at time of contact (i.e. age, sex, civil status, educational level, and ethnicity) [27]. The National Patient Register supplied data on diagnoses at time of contact, used for calculating the Charlson comorbidity Index (CCI) [28], while the Patient List Register provided the actual patient population per general practice.

We used the basic remuneration codes to identify clinic consultations (code 0101), CRP POCT (code 7120), and RADT POCT (code 7109) [24] in the NHISR. Patient characteristics were categorised: age groups (0–4, 5–9, 10–19, 20–39, 40–59, 60–79, 80+ years), CCI (0, 1, 2, 3+), civil status (single, other), highest level of education within household (0–10, 11–15, 16+ years), and ethnicity (Danish, western immigrant, non-western immigrant). As disease burden and diagnostic scope rapidly change in the early years of childhood, we defined smaller subgroups for youngest patients. As the provider identification number is on practice level, the number cannot be used to identify unique GPs, unless they work in a solo practice.

### Analyses

First, we described the distribution of patient characteristics for all clinic consultations, for consultations including use of CRPPOCTs, and for consultations with RADT POCTs use, both in daytime and in OOH general practice. Number of contacts and rate per 1,000 clinic consultations were calculated as an average per year and plotted with year on the X-axis. Next, the Bernoulli-distributed outcome POCT (yes, no) was analysed using generalised linear models from the Binomial family with a log-link, that is, binomial regression yielding proportion ratios (PRs) as the measure of association between patient characteristics and POCT use. We applied cluster-robust variance estimation at the practice level (i.e. provider) to accommodate the apparent clustering. Regression analysis was done in two pre-specified models; an unadjusted model and a model adjusted for age, sex, calendar time, CCI, civil status, level of education, and ethnicity. PRs with 95% confidence intervals (CI) were presented. Stata 14 (StataCorp LP, College Station, TX, USA) was used for analyses and EXCEL, Microsoft 365, for the figures.

## Results

### Study population

Table 1 presents the distribution of patient characteristics for all clinic consultations in daytime and OOH general practice, for all clinic consultations, consultations with use of CRP POCTs, and consultations with use of RADT POCTs. During the study period, 309.120.043 consultations were observed, approximately 295.8 million (95.7%) in daytime and 13.3 million (4.3%) OOH.

**Table 1.** Patient characteristics of study population, for all clinic consultations, consultations with CRP-, and consultations with RADT point-of-care test use, stratified for daytime and out-of-hours.

	Daytime			Out-of-hours		
	Consultations	Consultations with use of CRP tests	Consultations with use of RADT tests	Consultations	Consultations with use of CRP tests	Consultations with use of RADT tests
Total (*n*)	295,773,393	17,742,997	7,414,807	13,346,650	652,884	1,729,751
Sex (%)						
Women	58.7	59.4	58.5	52.9	56.3	54.7
Men	41.3	40.6	41.5	47.1	43.7	45.3
Age groups (in years) (%)						
0–4	4.7	4.1	14.0	22.1	13.9	27.4
5–9	3.6	2.6	12.6	9.8	5.6	15.7
10–19	8.1	8.6	21.4	13.2	12.7	18.5
20–39	22.1	22.4	30.7	26.6	29.0	27.0
40–59	27.0	28.8	15.5	18.2	23.3	9.4
60–79	27.9	28.0	5.3	8.8	13.8	1.9
80+	6.6	5.5	0.5	1.2	1.8	0.1
Charlson comorbidity Index (%)						
0	70.8	68.7	87.7	82.1	74.3	89.1
1	14.0	15.2	8.6	11.3	14.7	8.7
2	8.2	8.4	2.4	3.8	5.9	1.6
3+	7.0	7.7	1.2	2.8	5.0	0.7
Civil status (%)						
Single	28.8	27.1	16.0	17.9	21.9	11.1
Other	71.2	72.9	84.0	82.1	78.1	88.9
Highest level of education in household (in years) (%)						
0–10	24.2	23.2	15.8	21.6	22.1	16.7
11–15	47.8	48.7	48.9	49.7	49.6	50.2
16+	25.1	25.9	33.2	26.2	26.0	30.9
Missing	2.9	2.2	2.1	2.4	2.3	2.1
Ethnicity (%)						
Danish	89.9	90.7	88.5	88.0	88.0	87.2
Western immigrant	2.8	2.8	2.4	2.0	2.7	1.7
Non-western immigrant	7.4	6.5	9.1	9.9	9.4	11.1

The most frequent groups attending a consultation, both daytime and OOH, were women, patients without comorbidity, patients with 11–15 years of education in household and patients with Danish ethnicity. Compared with daytime care, a larger part of younger patients presented at the OOH service.

### Development of POCT use over time

**Figure 1.**
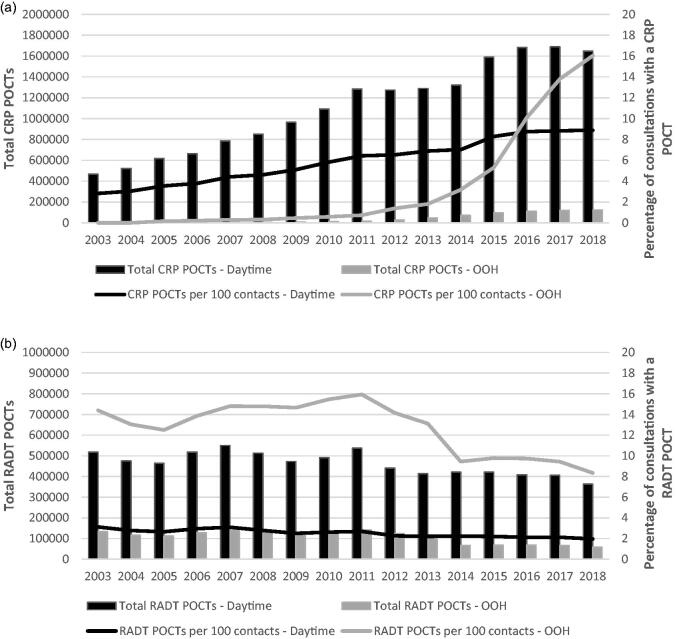
(a) Total and relative use of CRP per year, for daytime and out-of-hours primary care. Total usage is plotted as columns and relative usage as lines. (b) Total and relative use of RADT per year, for daytime and out-of-hours primary care.

The absolute use of CRP POCTs increased in the general practice setting between 2003 and 2018 (Figure 1(a,b)), peaking in 2017. From 2003 to 2018, the relative use increased from 2.8% to 8.9% of all daytime consultations and from 0.2% to 18.1% of all OOH consultations. Whereas the increase at daytime was almost constant, the use of CRP POCTs at the OOH steeply increased around 2013. The absolute use of RADT POCTs in both daytime and OOH general practice decreased between 2003 and 2018. In 2003, 3.1% of daytime and 14.4% of OOH consultations included the use of POCT RADT, while in 2018, this proportion was reduced to 2.0% and 8.3%, respectively.

### Patient characteristics associated with POCT use

We explored associations between patient characteristics and POCT use (Tables 2 and 3). Higher age (40–59 years, daytime: adj. PR = 1.02; OOH: adj. PR = 1.06; 60–79 years; OOH: adj.PR = 1.09) and existing comorbidity (daytime: adj. PR = range 1.04–1.10; OOH: adj.PR = range 1.12–1.15) were significantly associated with a higher use of CRP testing in daytime and OOH general practice. A significantly lower use of CRP testing was found for patients with higher educational level (daytime: adj.PR = range 0.96–0.99; OOH: adj.PR = range 0.96–0.98). Additionally, female patients, non-single patients, and ethnically Danes were associated with higher CRP POCT use.

**Table 2.** The crude and adjusted proportion ratios (PR), after regression analyses on cluster level (i.e., provider), of CRP use for patient- and contact characteristics, stratified for daytime and out-of-hours.

	Daytime		OOH	
	Crude PR	Adj. PR^a^	Crude PR	Adj. PR^a^
Sex				
Women	Ref	Ref	Ref	Ref
Men	0.97 (0.97; 0.98)	0.97 (0.96; 0.97)	0.87 (0.86; 0.88)	0.90 (0.89; 0.90)
Age groups (in years)				
0–4	0.85 (0.81; 0.90)	0.83 (0.79; 0.87)	0.58 (0.54; 0.62)	0.71 (0.67; 0.76)
5–9	0.74 (0.71; 0.76)	0.72 (0.70; 0.74)	0.52 (0.50; 0.54)	0.59 (0.57; 0.61)
10–19	1.04 (1.03; 1.06)	0.99 (0.98; 1.01)	0.88 (0.87; 0.89)	0.83 (0.82; 0.84)
20–39	Ref	Ref	Ref	Ref
40–59	1.06(1.05; 1.07)	1.02 (1.01; 1.03)	1.17 (1.16; 1.19)	1.06 (1.05; 1.08)
60–79	1.00 (0.98; 1.02)	0.90 (0.88; 0.92)	1.44 (1.41; 1.47)	1.09 (1.08; 1.11)
80+	0.83 (0.81; 0.86)	0.76 (0.74; 0.78)	1.34 (1.29; 1.38)	0.94 (0.91; 0.97)
Charlson comorbidity Index (CCI)				
0	Ref	Ref	Ref	Ref
1	1.12 (1.11; 1.13)	1.10 (1.09; 1.11)	1.44 (1.42; 1.47)	1.13 (1.12; 1.14)
2	1.06 (1.04; 1.07)	1.04 (1.03; 1.05)	1.72 (1.68; 1.76)	1.12 (1.11; 1.13)
3+	1.12 (1.11; 1.14)	1.10 (1.08; 1.11)	1.99 (1.94; 2.05)	1.15 (1.13; 1.17)
Civil status				
Single	Ref	Ref	Ref	Ref
Other	1.09 (1.08; 1.10)	1.12 (1.11; 1.13)	0.78 (0.76; 0.79)	1.09 (1.08; 1.10)
Highest level of education in household (in years)				
10	Ref	Ref	Ref	Ref
10-15	1.06 (1.05; 1.07)	0.99 (0.98; 0.99)	0.98 (0.96; 0.99)	0.98 (0.97; 0.99)
16+	1.07 (1.06; 1.09)	0.96 (0.94; 0.97)	0.97 (0.95; 0.99)	0.96 (0.94; 0.97)
Missing	0.79 (0.78; 0.81)	0.93 (0.92; 0.94)	0.93 (0.91; 0.95)	0.99 (0.97; 1.01)
Ethnicity				
Danish	Ref	Ref	Ref	Ref
Western immigrant	1.01 (1.00; 1.03)	0.96 (0.95; 0.98)	1.30 (1.27; 1.34)	1.02 (1.000; 1.04)
Non-western immigrant	0.87 (0.85; 0.90)	0.82 (0.80; 0.84)	0.94 (0.91; 0.98)	0.96 (0.93; 0.99)

^a^Adjusted for sex, age, Charlson Comorbidity Index, civil status, ethnicity, education, year, and month.

**Table 3.** The crude and adjusted proportion ratios (PR), after regression analyses on cluster level (i.e., provider), of RADT use for patient- and contact characteristics, stratified for daytime and out-of-hours.

	Daytime		OOH	
	Crude PR	Adj. PR^a^	Crude PR	Adj. PR^a^
Sex				
Women	Ref	Ref	Ref	Ref
Men	1.01 (1.00; 1.01)	1.02 (1.02; 1.03)	0.93 (0.92; 0.94)	0.93 (0.92; 0.93)
Age groups (in years)				
0–4	2.14 (2.10; 2.19)	1.89 (1.85; 1.93)	1.22 (1.18; 1.26)	1.06 (1.03; 1.10)
5–9	2.55 (2.52; 2.58)	2.34 (2.32; 2.36)	1.57 (1.56; 1.59)	1.42 (1.40; 1.44)
10–19	1.90 (1.89; 1.91)	1.84 (1.82; 1.85)	1.38 (1.37; 1.40)	1.34 (1.33; 1.35)
20–39	Ref	Ref	Ref	Ref
40–59	0.42 (0.41; 0.42)	0.43 (0.42; 0.43)	0.51 (0.51; 0.52)	0.52 (0.52; 0.53)
60–79	0.14 (0.14; 0.14)	0.16 (0.16; 0.16)	0.21 (0.21; 0.22)	0.25 (0.24; 0.25)
80+	0.05 (0.05; 0.05)	0.07 (0.07; 0.07)	0.08 (0.08; 0.09)	0.12 (0.11; 0.12)
Charlson comorbidity Index (CCI)				
0	Ref	Ref	Ref	Ref
1	0.50 (0.49; 0.50)	0.87 (0.87; 0.88)	0.71 (0.70; 0.71)	0.88 (0.87; 0.88)
2	0.24 (0.24; 0.24)	0.77 (0.76; 0.77)	0.38 (0.37; 0.39)	0.77 (0.76; 0.78)
3+	0.14 (0.14; 0.14)	0.61 (0.60; 0.62)	0.22 (0.21; 0.23)	0.61 (0.60; 0.63)
Civil status				
Single	Ref	Ref	Ref	Ref
Other	2.12 (2.09; 2.14)	1.17 (1.16; 1.17)	1.74 (1.72; 1.77)	1.21 (1.20; 1.22)
Highest level of education in household (in years)				
10	Ref	Ref	Ref	Ref
10-15	1.57 (1.55; 1.59)	1.16 (1.15; 1.17)	1.31 (1.30; 1.32)	1.18 (1.17; 1.19)
16+	2.03 (1.99; 2.06)	1.28 (1.26; 1.30)	1.52 (1.51; 1.54)	1.32 (1.30; 1.33)
Missing	1.14 (1.13; 1.16)	1.02 (1.01; 1.03)	1.13 (1.12; 1.15)	1.05 (1.04; 1.06)
Ethnicity				
Danish	Ref	Ref	Ref	Ref
Western immigrant	0.89 (0.88; 0.91)	0.98 (0.97; 1.00)	0.85 (0.83; 0.86)	0.95 (0.93; 0.96)
Non-western immigrant	1.25 (1.21; 1.29)	0.95 (0.92; 0.97)	1.12 (1.10; 1.14)	1.08 (1.06; 1.10)

^a^Adjusted for sex, age, Charlson Comorbidity Index, civil status, ethnicity, education, year, and month.

A significantly higher use of RADT testing was found for patients aged 0–19 years (daytime: adj.PR = range 1.84–2.34; OOH: adj.PR = range 1.06–1.42) and having higher educational level (daytime: adj.PR = range 1.16–1.28; OOH: adj.PR = range 1.18–1.32), whereas comorbidity was associated with a lower use of RADT testing (daytime: adj.PR = range 0.61–0.87; OOH: adj.PR = range 0.61–0.88). Additionally, male patients in daytime general practice, non-single patients, and non-western immigrants OOH were associated with higher CRP POCT usage.

## Discussion

### Principal findings

Overall, the use of RADT POCTs in Danish general practice decreased between 2003 and 2018 in daytime and OOH, whereas the use of CRP POCTs increased. This development over time was particularly evident at the OOH services. The use of CRP testing was positively associated with patients being aged 40–59 years (additionally 60–79 years OOH) and having existing comorbidity. Contrary, patients with a higher level of education were less often tested with a CRP POCT. A higher use of RADT testing was found for young patients aged 0–19 years and patients with higher education level in the household, whereas a lower use was found for patients with comorbidity.

### Strengths and limitations

This study is based on a large nationwide dataset, including all general practice contacts in Denmark during sixteen years. Danish national registers are considered as valid data sources for research. However, when interpreting the results of this study several limitations must be kept in mind.

First, data based on standard remuneration coding is useful for research purposes, but some reservations may exist as validity has not been studied [26]. Secondly, results should of course always be interpreted with caution without direct clinical information [26–29]. Thirdly, we used hospital-based data to calculate comorbidity, which may have led to underestimation of comorbidity [30], as patients with mild chronic diseases are often treated solely in general practice. Lastly, our study design and available data did not allow us to explore clinical indications for performing either a CRP and/or a RADT test, neither the GPs’ clinical reasoning.

### Comparison with existing literature

The observed increase in the use of CRP testing in Danish general practice over time has also previously been documented in another Danish study [20]. Haldrup et al. found an increase of 132% between 2004 and 2013 including both daytime practices and OOH services. In line with the findings of our study, Haldrup et al. also demonstrated a decline of 8.6% in the use of RADT tests. The increased use of CRP POCTs seems to be in line with its enrolment in general practice, with the steeper increase in the OOH setting due to later introduction. Changes in the organisation of care in Danish general practice, with an increased use of nurses assisting in clinic consultations, may also have contributed to the general increase in the use of CRP tests [31]. The higher use of CRP POCTs in the OOH setting can, at least partly, be explained by differences in patient populations, with many acute infections presented at the OOH services [3,32]. Furthermore, the challenging conditions at the OOH services, with no previously established GP-patient relationship, may also contribute to a higher use of CRP POCTs.

The decline in the use of RADT POCTs could reflect a more restrictive approach, as its relevance and indication for use is debated in relation to antibiotic prescribing [6,20]. The high use of RADT testing for children aged 0–9 years compared to other age groups could be explained by the high incidence of acute upper respiratory tract infections among children [3,^4^]. Also, the modified Centor Score includes a recommendation for testing children aged between 3 and 14 years more frequently, due to a higher risk of having an infection caused by group A Streptococci.

### Implications for research and practice

The past decades, the use of CRP POCT in both daytime and OOH general practice settings increased, whereas the use of RADT POCT decreased. In some practices, patients with symptoms of an acute respiratory tract infection are tested prior to a consultation with a comprehensive history taking and clinical examination [25]. Perhaps we have reached a time where use of POCTs not necessarily always reduces the use of antibiotics. To clarify how and when POCTs are used most optimally, more knowledge about the variation in use of POCTs between GPs and practices, and its relation with antibiotic use is needed.

## Ethical approval

According to Danish law, approval from the Committees on Health Research Ethics was not needed as the study included no biomedical intervention. The project is listed in the record of processing activities at the Research Unit for General Practice in Aarhus in accordance with the provisions of the General Data Protection Regulation (GDPR).
